# Smart heating sleeping bags for improving wearers' thermal comfort at the feet

**DOI:** 10.1186/2046-7648-4-S1-A92

**Published:** 2015-09-14

**Authors:** Chengjiao Zhang, Dandan Lai, Yehu Lu, Faming Wang, Kalev Kuklane

**Affiliations:** 1 Laboratory for Clothing Physiology and Ergonomics (LCPE), the National Engineering Laboratory for Modern Silk, Soochow University, Suzhou, 215123 China; 2 Thermal Environment Laboratory, Department of Design Sciences, Lund University, Lund, Sweden

## Introduction

Sleeping bags are portable products, which are essential for sleeping outdoors. Generally, a sleeping bag is comprised of an outer layer, an inner lining layer and the filler. The EN 13537 (2012) and ASTM F1720 (2014) standards are widely used to determine the thermal insulation of sleeping bags by means of thermal manikin. Previous studies have found that local thermal discomfort at feet was often seen despite the mean skin temperature was well within thermoneutral range under the defined comfort and limit temperature [1]. Thus, it is meaningful to design smart heating sleeping bags to improve the local thermal comfort of the users. In this study, a novel smart sleeping bag was developed by incorporating heating fabrics into the feet region of the bag. The physiological and psychological responses when using traditional sleeping bag and the smart sleeping bag were investigated and compared.

## Methods

Two smart sleeping bags used at different temperatures were developed. The thermal insulation of those two sleeping bags, Vau (V) and Mar (M), were first measured by a thermal manikin 'Newton' (Measurement Technology Northwest, Seattle, USA) according to EN 13537 (2012) [2]. For each bag, two test scenarios were selected: heating off (i.e., CONTROL) and heating on (i.e., ON). The heating power was set at 20 W. Eight subjects (four females) participated in this study. The test duration was 3 hours. The comfort temperature of the sleeping bags V and M was 5.5 °C and -0.5 °C, respectively. The limit temperature was 0.5 °C and -6.4 °C, respectively. The RH and wind speed for all tests were 80% (5%) and 0.4 (0.1) m.s^-1^, respectively. Subjective perceptions (e.g., whole body and local thermal sensation, comfort sensation and skin wetness) were collected 15 min before the trial, the beginning of the trial, the 20th min and the 180th min of the trial. Physiological responses, i.e., oxygen consumption, blood flow, and heart rate were measured throughout each human trial. The mean skin temperature (T_sk_) was calculated by the Gagge and Nishi's 8-point equation.

## Results

For the CONTROL, it was found that the temperatures at the left fourth toe and the left foot fell continuously linear dropping throughout the 3-hour testing period, for male, the mean toe temperature declined from 25.5 °C and 24.9 °C to 14.6 °C and 15.4 °C for V and M, respectively; for female, the mean toe temperature decreased from 25.3 °C and 25.1 °C to 17.6 °C and 18.2 °C for V and M, respectively. The mean foot temperature has the similar tendency with that of toes. Besides, strong cold and uncomfortable feeling at feet was observed for most subjects. However, the T_sk _well stayed within the thermoneutral range (i.e., 32-34 °C) throughout the whole test. For all testing scenarios with heating on (i.e., ON), the toe and foot temperatures of all subjects were well maintained above 22.0 °C and 30.0 °C, respectively. The local thermal and comfort sensation denoted that subjects were in thermal neutral conditions.

## Discussion

This laboratory study validated that the introduction of the heating fabric could improve the subject's thermal and comfort sensation. It has proven that the smart heating sleeping bag could keep the users foot and toe remaining comfort throughout the testing period under defined temperature. Besides, the newly developed smart sleeping bags could significantly improve local thermal comfort under the defined wear temperature. However, it was evident that heating at foot region has no obvious effect on the T_sk_. This study also confirmed again that the EN 13537 (2012) defined temperature only focus the whole body thermal balance, with less regard on the extremities (i.e., the feet and toes).
